# Metabolomic profile of acute myeloid leukaemia parallels of prognosis and response to therapy

**DOI:** 10.1038/s41598-023-48970-0

**Published:** 2023-12-09

**Authors:** Lukasz Bolkun, Tomasz Pienkowski, Julia Sieminska, Joanna Godzien, Karolina Pietrowska, Janusz Kłoczko, Agnieszka Wierzbowska, Marcin Moniuszko, Mariusz Ratajczak, Adam Kretowski, Michal Ciborowski

**Affiliations:** 1 Department of Hematology, Medical University of Bialystok, 15-276 Bialystok, Poland; 2 Clinical Research Centre, Medical University of Bialystok, M. Sklodowskiej-Curie 24A, 15-276 Bialystok, Poland; 3 Department of Hematology, Medical University of Lodz, 93-513 Lodz, Poland; 4 Department of Regenerative Medicine and Immune Regulation, Medical University of Bialystok, Bialystok, Poland; 5 Department of Allergology and Internal Medicine, Medical University of Bialystok, Bialystok, Poland; 6 Stem Cell Institute at James Graham Brown Cancer Center, University of Louisville, Louisville, KY USA; 7 Department of Endocrinology, Diabetology and Internal Medicine, Medical University of Bialystok, 15-276 Bialystok, Poland

**Keywords:** Acute myeloid leukaemia, Cancer metabolism, Biomarkers, Cancer screening, Tumour biomarkers

## Abstract

The heterogeneity of acute myeloid leukemia (AML), a complex hematological malignancy, is caused by mutations in myeloid cells affecting their differentiation and proliferation. Thus, various cytogenetic alterations in AML cells may be characterized by a unique metabolome and require different treatment approaches. In this study, we performed untargeted metabolomics to assess metabolomics differences between AML patients and healthy controls, AML patients with different treatment outcomes, AML patients in different risk groups based on the 2017 European LeukemiaNet (ELN) recommendations for the diagnosis and management of AML, AML patients with and without FLT3-ITD mutation, and a comparison between patients with FLT3-ITD, CBF-AML (Core binding factor acute myelogenous leukemia), and MLL AML (mixed-lineage leukemia gene) in comparison to control subjects. Analyses were performed in serum samples using liquid chromatography coupled with mass spectrometry (LC–MS). The obtained metabolomics profiles exhibited many alterations in glycerophospholipid and sphingolipid metabolism and allowed us to propose biomarkers based on each of the above assessments as an aid for diagnosis and eventual classification, allowing physicians to choose the best-suited treatment approach. These results highlight the application of LC–MS-based metabolomics of serum samples as an aid in diagnostics and a potential minimally invasive prognostic tool for identifying various cytogenetic and treatment outcomes of AML.

## Introduction

Metabolomics studies are based on disturbances in the abundances of total metabolites, such as lipids and fatty acids, in an analyzed sample (such as body fluids or tissues from tumors for in situ analysis of metabolic changes). These alterations in whole metabolic compositions occur due to tumor proliferation, which requires the intake of basic building blocks for cell growth, or the release of oncometabolites. The process is described thoroughly by the Warburg effect. Tumors take up free lipids from the bloodstream to incorporate them into their own cell membrane as they grow. Thus, metabolomics, as a fairly new field of science, has the potential to discover metabolic pathways disturbed by a particular disease and subsequently propose novel discriminative or diagnostic markers and therapy targets^1,2^. Despite a multitude of efforts, the continued search for markers of acute myeloid leukemia (AML) is crucial for the identification of metabolic pathways involved in either the promotion or inhibition of AML cell growth, progression of AML and susceptibility of AML cells to cytotoxic drugs. Changes in metabolites, lipids and small molecules involved in metabolic processes identified via gas/liquid chromatography–mass spectrometry (GC/LC–MS) and assessed by multivariate statistical analysis may present us with discriminative phenotypic profiles of distinct AML alterations and stages. Some studies have attempted to examine global changes in metabolites to identify biomarkers associated with phenotypic changes in various cancers^1,3^. However, there are very few AML studies that involve metabolomics^4–7^. The closest research were conducted on bone marrow^4^ and bone marrow serum samples^7^. Both studies utilize metabolomics profiling, yet they employ methodologies distinct from ours. The first study relies on HRMAS-NMR–based metabolomics, while the latter employs GC–TOF–MS analysis. Nonetheless, both studies offer significant insights from perspectives differing from our own.

In the present study, we used an LC–MS metabolomics approach to examine global metabolic profiles in serum samples of AML patients by evaluating small molecules compositions in patients and healthy controls; evaluating differences in metabolomic profiles in patients with complete recurrence (CR) and no recurrence (NR); assessing metabolomic differences between AML patients with different cytogenetic risk; and investigating metabolomic differences in patients with or without FLT3-ITD gene mutation; we also carried out a comparison between patients with FLT3-ITD, CBF-AML (Core binding factor acute myelogenous leukemia), and MLL AML (mixed-lineage leukemia gene) in comparison to control subjects.

## Materials and methods

### Patient cohort and classification

All experimental procedures were approved by the ethics and research committee of the Medical University of Bialystok, Poland, and were performed in accordance with institutional guidelines at Bialystok University Hospital (R-I-002/393/2018). After obtaining informed consent, a total of 100 serum samples collected from previously untreated acute non-promyelocytic leukemia patients were included in the study (Table 1). The median age of the patients at the time of sample collection was 50, and the range was 18–67. Forty-five subjects were female, and fifty-five were male. Diagnoses were established following the WHO classification system^8^. Blood counts and flow cytometry were performed to confirm the presence of blastic cells, whereas cytogenetic and molecular studies, including FISH (AML1/ETO, CBFß/MYH11, MLLT3-MLL and frequently mutated genes FLT3-ITD, NPM1, CEBPA, TP53), were carried out to determine the risk group of the patients according to WHO recommendations. Participants included in the study were free from autoimmune, infectious, or metabolic diseases such as dyslipidemia and diabetes. Additionally, none of the control subjects had a history of neoplasia. Serum samples were collected before treatment from all included AML patients and after treatment from those AML patients with complete remission (CR) in the fasting state (in the morning).

**Table 1:** Characteristics of the AML patients.

Number of patients	100
Mean (range) age, year	50 (18–67)
Mean (± SD) white blood cell count (G/l)	38.12 (2.0–212.3)
Mean (range) the percentage of the blastic cells in peripheral blood	47 (0–98)
Mean (range) of blastic cells percentage? in bone marrow	56 (20–93)
1. Acute myeloid leukemia with recurrent genetic abnormalities	*n* (%)
*t*(8;21) (q22;q22);(AML1/ETO)	8 (8%)
inv(16) (p13;q22) or *t*(16;16) (p13;q22); (CBFß/ MYH11)	6 (6%)
*t*(9;11); MLLT3-MLL	9 (9%)
*NPM1*^wt^*FLT3*^low^/*NPM1*^mut^*FLT3*^wt^	5 (5%)
*NPM1*^wt^*FLT3*^high^	15 (15%)
*Biallelic CEBPA*_*mut*_	1 (1%)
2. AML with multilineage dysplasia without antecedent MDS	10 (10%)
3. AML therapy-related	0 (0.0%)
4. AML not otherwise categorized (FAB classification)	n (%)
AML, minimally differentiated	5 (5%)
AML without maturation	10 (10%)
AML with maturation	10 (10%)
Acute myelomonocytic leukaemia (AMMoL)	9 (9%)
Acute monoblastic leukaemia	7 (7%)
Acute monocytic leukaemia	5 (5%)
DAC	51
DA	49
The outcome of induction therapy DAC or DA	
CR achieved after the first induction, n, %	50 (50%)
Favourable risk	15 (15.0%)
Intermediate I and II risk	50 (50.0%)
Unfavourable risk	45 (45.0%)

*AML* acute myeloid leukaemia, *CR* complete remission, *NPM1*_*mut*_ mutated nucleophosmin, biallelic *CEBPA*_*mut*_ mutated core-binding factor leukaemia, *FLT3-ITD* internal tandem duplication of Fms-like tyrosine kinase 3.

Patients were classified based on risk as follows: 15 patients had good risk (8 patients with *t*(8;21), 6 with inv(16) and one with mutated core-binding factor leukemia (CEBPA_*mut*_)), 50 subjects had intermediate risk (including patients with diploid karyotype features, 5 patients with both mutated nucleophosmin (NPM1_*mut*_) and internal tandem duplication of Fms-like tyrosine kinase 3 (FLT3-ITD) and 15 patients with FLT3-ITD without NMP1_*mut*_), and 45 subjects were classified as having unfavorable risk (with del(5q), del(7q) or complex (≥ 3) abnormalities).

AML patients were treated in the Haematology Department of the University Hospital of Bialystok from 2012 to 2019 with 7-day induction chemotherapy regimens corresponding to the standard therapy based on the Polish Adult Leukaemia Group; notably, the treatment schedules depended on the preferences of the center or the hematologist. Briefly, cytarabine was delivered as a continuous IV infusion for seven consecutive days at a dose of 200 mg/m^2^, while anthracycline was delivered for three consecutive days as an IV push at a dose of 60 mg/m^2^ in a DA schedule. Additionally, cladribine was administered for 5 days as an IV push at a dose of 5 mg/m^2^ in DAC schedule^9^. After induction, the morphological response was evaluated following the recommendation of Chason et al*.*^10^; among the selected samples, CR was observed in 50 patients after the first induction, and 50 patients were nonresponders (NR).

We performed rigorous age- and gender-matching between the patients diagnosed with AML and the healthy control group, meticulously ensuring that any potential influences from age and gender variables were effectively minimized in our study. The control group was population-based and comprised twenty age-matched healthy volunteers (10 males, 10 females). Samples were collected from the subjects if they were free of fever for 1 week, not receiving any medications, not being pregnant and did not have a history of any chronic diseases.

### Experimental methods

The experimental methods are described in the Supplementary Materials.

## Results

In this study, we obtained the metabolic profiles of 120 serum samples collected from 100 AML patients and 20 healthy controls (Table 1). We then used these profiles to evaluate several comparisons: metabolomic differences between AML patients and controls; metabolomic differences between patients based on the type of response to induction therapy, namely, CR or NR; metabolomic differences before and after induction in a group of responders; metabolomic differences between patients in different risk groups according to the 2017 ELN recommendations for the diagnosis and management of AML^11^; and metabolomic differences between AML patients with and without FLT3-ITD mutation.

### Differences between AML patients and controls

The first comparison identified the metabolomic differences between AML patients and healthy controls. Metabolic pathway impact analysis showed differences mostly in the lipid profiles, especially of glycerophosphocholine (PC), lysoglycerophosphocholine (LPC) and sphingomyelin (SM) (Supplementary Fig. 1A). However, not only lipids but also singular metabolites in the serum samples were affected. In comparison to healthy controls, AML patients had an increased abundance of sterol sulfate conjugate (ST) (21:1;O2;S) of 259%, PC (28:0) of 238%, amino-hexadecanoic acid of 107%, acyl carnitine (AC) (8:0) of 87%, and cortisol of 84%. Additionally, the tricarboxylic acid (TCA) metabolite citric acid and PC (34:5) in AML patients compared to healthy controls were decreased by 98% and 83%, respectively (Supplementary Table 2). An OPLS-DA model clearly separated the AML patients from the controls, as shown in Supplementary Fig. 2A and B in the Supplementary Materials. Models based on both LC–MS ionization modes present a clear separation of the studied groups with good R^2^ and Q^2^ values (Supplementary Materials).

### Differences between types of responses (CR vs.* NR)*

The second comparison focused on the possible impact on metabolome in types of responses (CR, n = 50, NR, n = 50) to the applied regimen. Metabolic pathway impact analysis showed that the most affected pathway was glycerophospholipid metabolism (Supplementary Fig. 1B), as in the second comparison. The most affected SM (d18:1/12:0) was elevated by 80% in patients with CR compared to NR (Supplementary Table 3). OPLS-DA models were built to distinguish these groups and, as shown in Supplementary Fig. 3A and B in the Supplementary Materials, CR group is separated from the NR group. Models for both ionization modes data present a clear separation of the studied groups with good R^2^ and Q^2^ values (Supplementary Materials).

Additionally, we attempted to analyze differences between the DA and DAC approaches in ChT of AML. However, we were unable to obtain any statistically significant results for differences in successful treatment outcomes. Thus, the results of this comparison are not included.

### Differences between ELN risk groups

Another comparison evaluated different ELN risk groups, namely, those with favorable (group 1), intermediate (group 2) or adverse (group 3) risk, stratified based on the 2017 ELM recommendations.

**Figure 1:**
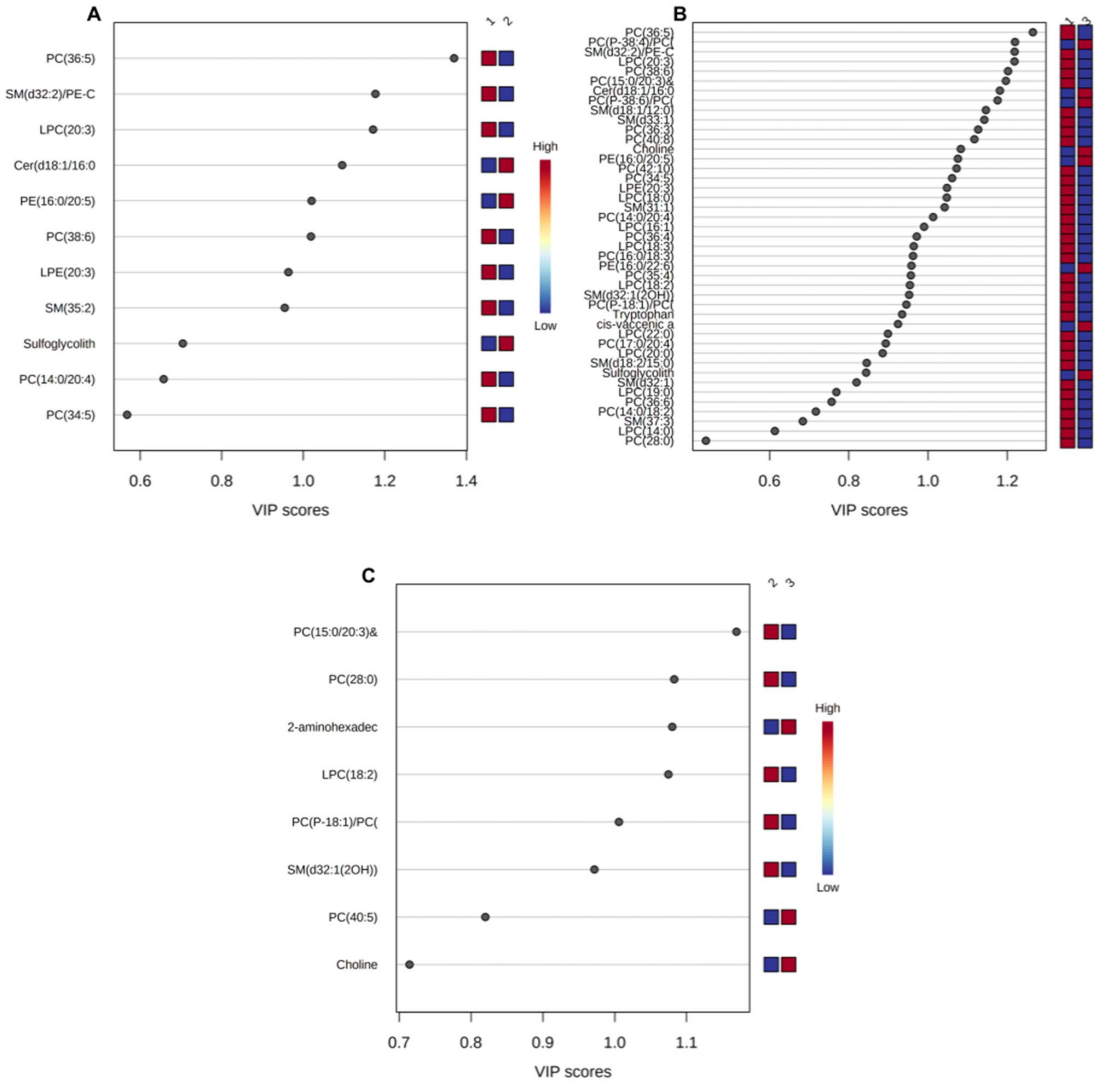
Importance in Projection (VIP) plot of metabolites contributing to group separation between (**A**) AML patients and controls. (**B**) Before and after treatment. (**C**) CR and NR. The VIP plot illustrates the most significant metabolite features identified through OPLS-DA. The colored boxes on the right side indicate the relative concentration of corresponding metabolites in serum samples. VIP is a composite score that considers the OPLS-DA loadings' squared sums, incorporating the degree to which the Y-variable is explained in each dimension. CR, Complete recurrence; NR, No recurrence.

For the first comparison (group 2 vs. group 1), metabolic pathway impact analysis showed that the most affected pathway was glycerophospholipid metabolism (Supplementary Fig. 4A). Overall, there were 11 dysregulated compounds (Fig. 1A). Three of them were significant and represented PCs (34:5, 36:5, and 38:6) However, PE (16:0/20:5) and glycolithocholate sulfate, which increased in abundance by 226% and 110%, respectively, exhibited nonsignificant increases (Supplementary Table 4).

For the second comparison (group 3 vs. group 1), glycerophospholipid metabolism and sphingolipid metabolism (Supplementary Fig. 4B) were affected. Overall, there were 43 dysregulated compounds (Fig. 1B). The metabolites that experienced the most significant impacts were PCs (28:0) and (34:5), showing reductions of 99% and 84% in group 3 in comparison to group 1, respectively. On the other hand, PE (16:0/20:5), choline, and Cer (d18:1/16:0) displayed notable increases of 360%, 88%, and 83% in group 3 in comparison to group 1, respectively (Supplementary Table 5).

For the third comparison (group 3 vs. group 2), glycerophospholipid metabolism (Supplementary Fig. 4C) was the most affected metabolic pathway with 8 dysregulated compounds overall (Fig. 1C). Despite PCs being the most altered, 2-aminohexadecanoic acid had the greatest increase in abundance of 171% in group 3 in comparison to group 1 (Supplementary Table 6). When comparing the differences between these ELN risk groups, the most discriminating compounds were lipids (Supplementary Fig. 4A–C).

OPLS-DA (Supplementary Fig. 5A–F) models were built to distinguish groups based on both ionization modes data, and the results indicated clear group separation with good R^2^ and Q^2^ values (Supplementary Materials).

### Differences between the patients with and without FLT3-ITD mutation

**Figure 2:**
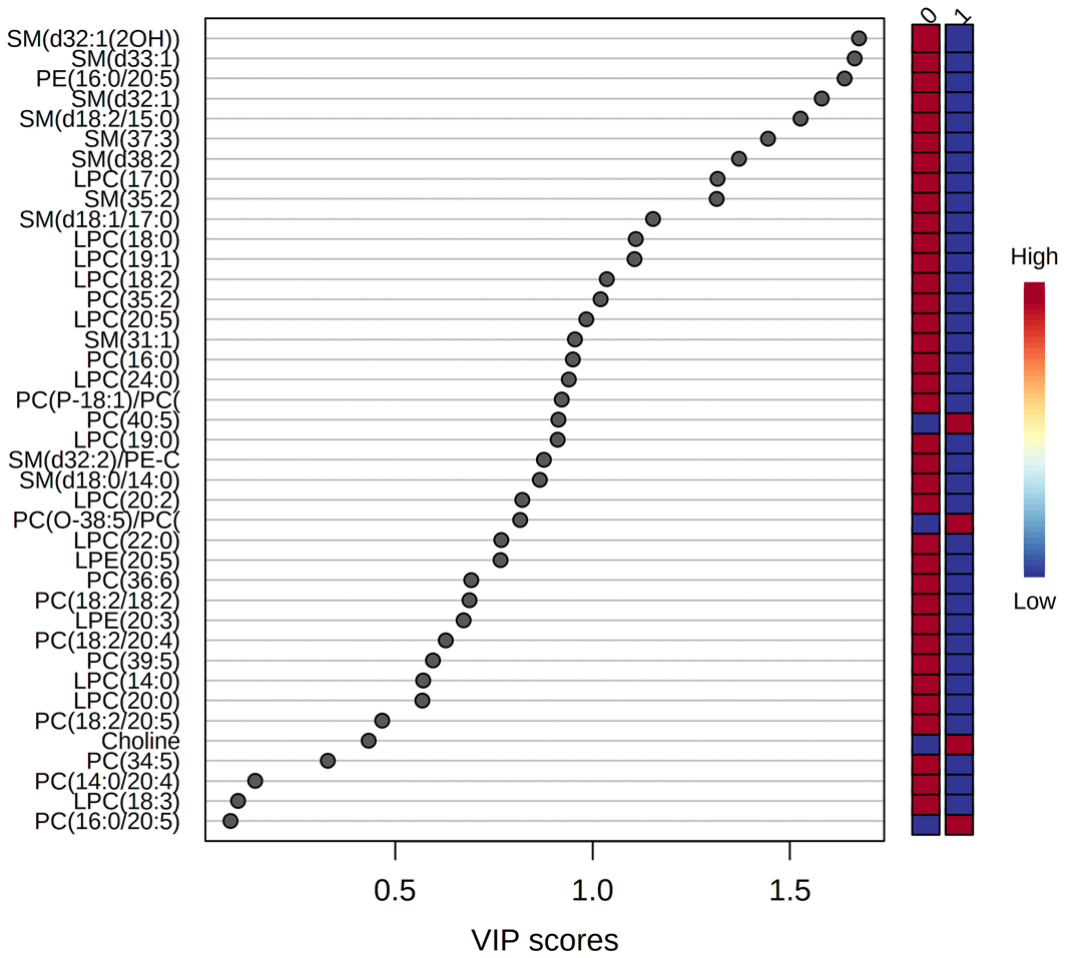
Importance in Projection (VIP) plot of metabolites contributing to group separation between patients with FLT3-ITD mutation and those with FLT3-WT. The VIP plot illustrates the most significant metabolite features identified through OPLS-DA. The colored boxes on the right side indicate the relative concentration of corresponding metabolites in serum samples. VIP is a composite score that considers the OPLS-DA loadings' squared sums, incorporating the degree to which the Y-variable is explained in each dimension.

This comparison assessed metabolomic differences between the patients with (n = 20) and without (n = 21) FLT3 mutation. Metabolic pathway impact analysis showed that the most affected pathway was glycerophospholipid metabolism (Supplementary Fig. 6). Overall, there were 40 dysregulated compounds. When comparing AML patients with and without FLT3 mutation, the most discriminating compounds were lipids (Fig. 2). The most affected lipid was SM (d33:1), which was decreased by 80.3% in patients with FLT3 mutation in comparison to patients without FLT3 mutation (Supplementary Table 7). OPLS-DA models were built for both ionization modes data, and the obtained results are shown in the Supplementary Materials (Supplementary Fig. 7A and B), indicating clear group separation with good R^2^ and Q^2^ values (Supplementary Materials).

### Differences between the patients with chromosomal aberrations of AML and control subjects

For deeper investigation of ELM classification, we conducted comparisons focused on the most common chromosomal aberrations in AML in comparison to healthy subjects. These abnormalities encompassed FLT-ITD, CBF, and MLL. In both comparisons of AML patients with FLT-ITD mutation and CBF-AML with the control group, the most distinguishing compounds were found within sphingolipid and glycerophospholipid metabolism (Supplementary Fig. 8A and B). For MLL-AML patients, the differentiating factors were related to glycerophospholipid metabolism and amino acid metabolism (Supplementary Fig. 8C).

In the patient group with FLT-ITD mutation, the most pronounced alterations were observed in PCs ((20:0) increased by 159%, and (34:5) decreased by 90%). Moreover, there were notable increases in sterols (ST 24:1;O4;S and ST 19:2;O3;S) by 128% and 86%, respectively, along with an 84% rise in cortisol levels (Supplementary Table 8). In the case of CBF-AML patients, distinct differences compared to controls were noted and displayed a heightened abundance of various molecules, including PCs ((35:2) by 255% and (38:6) by 147%), sterol (ST 24:1;O4;S by 158%), SM ((d18:2/14:0) by 143%), and LPC ((24:0) by 134%). Citric acid levels were notably reduced by 98% (Supplementary Table 9). In the context of MLL-AML, significant differences were observed in elevated abundance of PCs ((P-38:4)/PC(O-38:5) by 252%), sterol (ST 24:1;O4;S by 154%) and cortisol (by 105%), with decreased abundance of citric acid (by 97%) and PC ((34:5) by 92%) (Supplementary Table 9).

**Figure 3:**
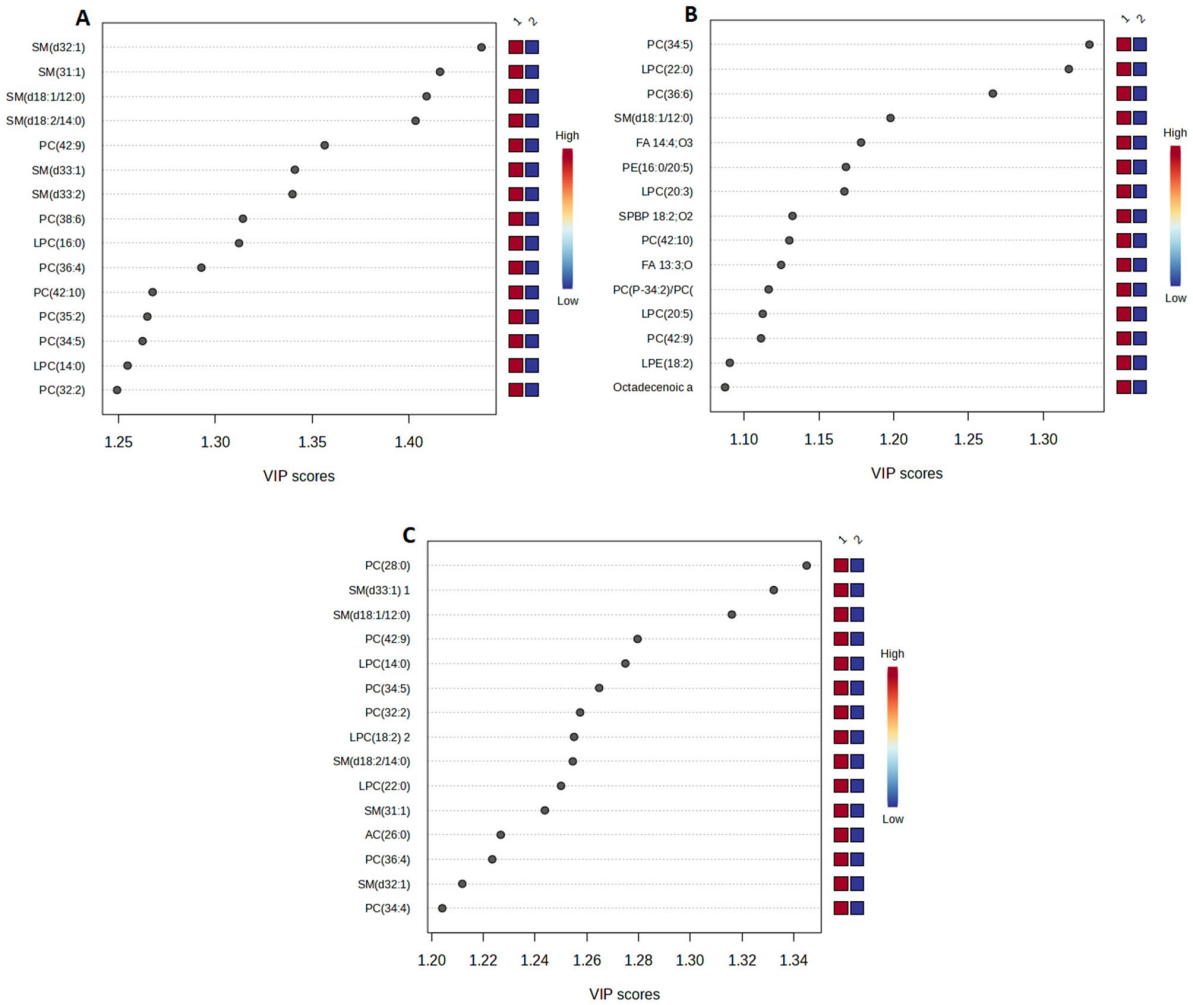
Importance in Projection (VIP) plot of metabolites contributing to group separation between patients with distinct genetic subgroups in comparison to control group. (**A**) FLT3-ITD to control group; (**B**) CBF-AML to control group; (**C**) MLL to control group. The VIP plot illustrates the most significant metabolite features identified through OPLS-DA. The colored boxes on the right side indicate the relative concentration of corresponding metabolites in serum samples. VIP is a composite score that considers the OPLS-DA loadings' squared sums, incorporating the degree to which the Y-variable is explained in each dimension. CBF-AML, Core binding factor acute myelogenous leukemia; MLL, Mixed-lineage leukemia gene.

When making comparisons between FLT-ITD patients and the control group, the most discriminative compounds that stood out were specific types of SMs: (d32:1), (31:1), (d18:1/12:0), and (d18:2/14:0) (Fig. 3A). In the case of CBF-AML patients compared to the control group, the compounds that exhibited the most discriminating value were certain types of PCs: (34:5) and (36:6), along with LPC (22:0) (Fig. 3B). Lastly, when analyzing AML patients with and without MLL in comparison to the control group, the discriminative compounds were PC (28:0) and SMs ((d33:1) and (d18:1/12:0)) (Fig. 3C).

OPLS-DA models were constructed to differentiate these categories (Supplementary Fig. 9A–F). In both ionization modes, the data exhibited distinct segregation among the investigated groups, as evidenced by favorable R2 and Q2 values (Supplementary Materials).

## Discussion

AML is aggressive and the most commonly occurring hematological malignancy characterized by clonal proliferation of myeloid cells at various stages of maturation^12,13^. Despite advances in diagnosis, stratification and treatment, the disease remains largely incurable with a high percentage of relapses, which causes a 5-year life expectancy not exceeding 25–30%^12–14^. AML is a heterogeneous disorder characterized by the accumulation of gene alterations, along with translocations and inversions, that contribute to disease biology and prognosis and, most importantly, carry prognostic importance. The discovery of novel discriminative biomarkers remains of utmost importance to provide new outcome definitions and therapeutic targets. Despite a multitude of efforts, the identification of crucial pathways and proteins involved in either promotion or inhibition of cell growth, progression of AML and susceptibility of AML cells to cytotoxic drugs is still warranted.

The repurposing of cellular energy metabolism is by no means the only metabolic trait that cancers can express. Another metabolic idiosyncrasy that cancer cells use to their advantage is the production of so-called oncometabolites. Indeed, lower plasma levels of cholesterol, lipids, and unsaturated fatty acids have been found in AML mice in comparison to healthy controls^15,16^, highlighting an involvement of fatty acid metabolism in AML, possibly related to the demand for lipids and cholesterol in tumor proliferation. The study of the mechanisms underlying the relationship between oncometabolites and tumor repopulation is fundamental for identifying efficient anticancer therapeutic strategies and novel serum biomarkers to overcome cancer relapse.

Thus, in this research, we focused on metabolites that can differentiate AML status based on specific conditions, namely, treatment outcome (CR and NR), treatment regimen in patients with CR, risk classification, and presence or absence of FLT3-ITD mutation.

**Figure 4:**
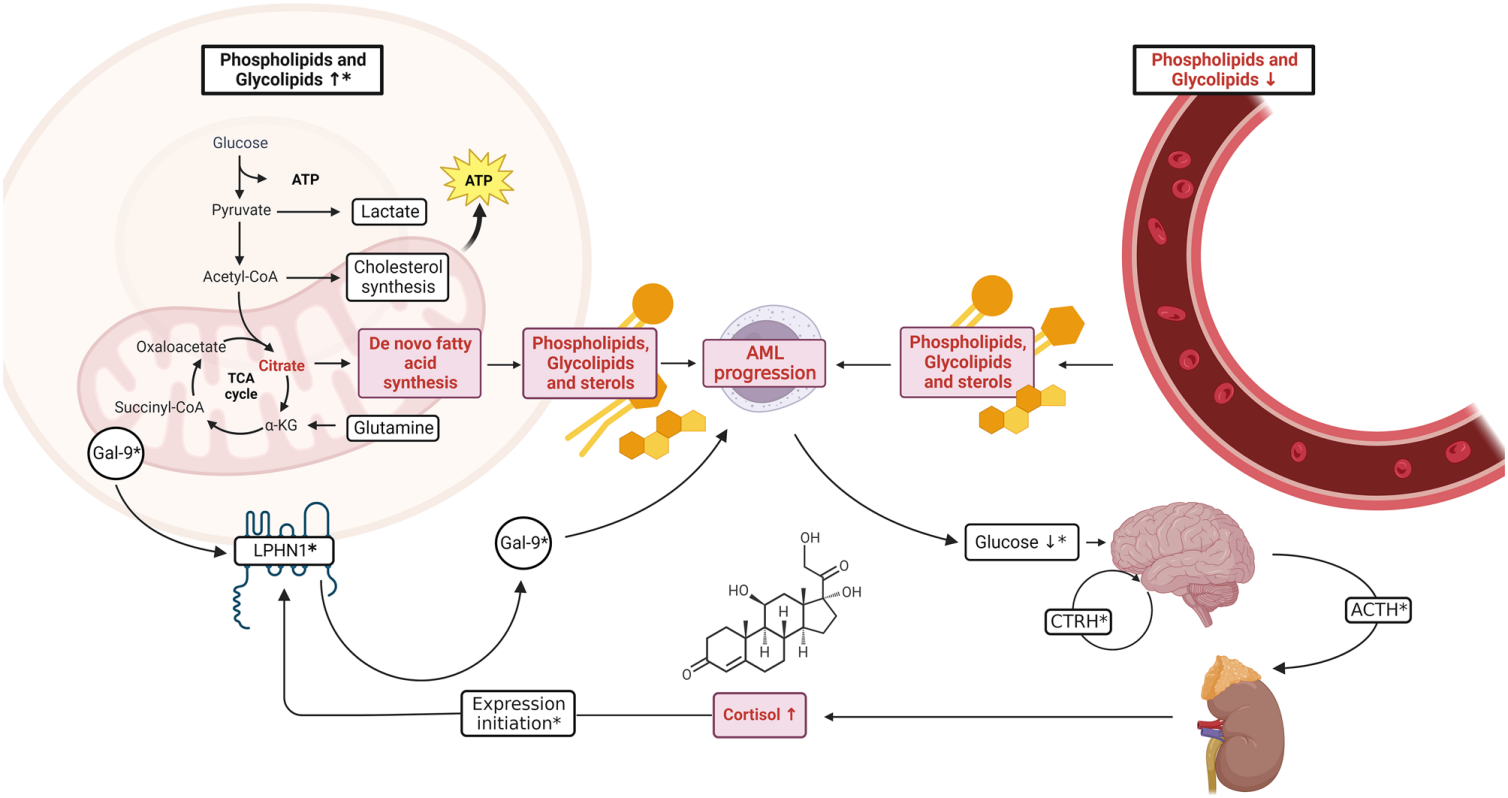
Main findings summary. White boxes depict data that falls beyond the scope of this research but holds relevance. White boxes or circles marked with “*” indicate literature data, while red boxes represent the primary discoveries of this study. Upward and downward arrows symbolize changes in the abundance of specified compounds. Arrows pointing in specific directions illustrate the progression of biochemical pathways. Gal-9, Galectin-9; CTRH,corticotropin-releasing hormone; ACTH, Adrenocorticotropic hormone. Created with BioRender.com.

The key to understanding metabolomic changes was to first research the differences between AML patients and healthy controls, and this was our starting point for making further comparisons. The alterations uncovered in this research were mostly lipid- and TCA-based. The composition of decreased PCs, LPCs, SMs and PEs is quite common for malignancies due to the Warburg effect and the necessity of extracellular lipid intake for tumor growth (Fig. 4). Our findings are consistent with the conclusions drawn by Lo Presti et al.^4^ concerning elevated PEs levels in bone marrow cells. This phenomenon, commonly observed in cancers, is associated with an increase in cellular PC levels, indicating heightened cellular proliferation. The most decreased PC was (34:5) by 83% and citric acid by 98% in comparison to healthy controls. The results for citric acid in AML patients seemed to be in accordance with Zeng et al.’s^17^ research based on a metabolomics investigation of TCA cycle alterations. The overexpressed IDH-2 gene in AML promotes leukemia cell survival and proliferation in vitro as well as in vivo through active IDH-2-mediated conversion of alpha-ketoglutarate to isocitrate/citrate to facilitate glutamine utilization for fatty acid (FA) synthesis. FA, which was also decreased in serum, is essential for the proliferation of leukemia cells. In conclusion, the increased demand of tumor cells for basic building components of cell membranes and their substrates for their precursors by extra- and intracellular means may lead to a decreased abundance of these components in serum. Lo Presti et al.^4^ recently brought attention to notable discrepancies in the levels of other metabolites among patients with IDH mutations—amino acids, indicating that the status of IDH has implications for multiple metabolic pathways. In fact, we also detected certain disruptions in amino acid metabolism (Supplementary Table 2). Additionally, Jones et al.^18^ determined that amino acids are metabolized in the TCA cycle in leukemic stem cells (LSCs). They further revealed that LSCs derived from individuals with de novo AML exhibit a specific reliance on amino acid metabolism for oxidative phosphorylation. In our findings, a reduction in amino acids was observed in the serum (Supplementary Table 2). Conversely, existing literature indicates an elevated presence of amino acids within cell lines^18^. Nonetheless, as discussed earlier, this contrast could be attributed to the Warburg effect, where LSCs consume essential cellular building blocks, potentially leading to their depletion in the serum. Interestingly, concerning the variations in fatty acid levels, Jones et al.^18^ also made an intriguing observation. When examining LSCs from relapsed patients, they identified a notable adaptation: the LSCs developed the capability to counteract the loss of amino acids by increasing their fatty acid metabolism. This phenomenon was not witnessed in LSCs from treatment-naive patients.

Additionally, fewer changes were observed in the SM and ceramide serum composition of AML patients. SM has been reported to be related to vital cell activities, such as growth, adhesion and survival^19^. Additionally, ceramides are correlated with cellular responses^20^ and are mostly found in high concentrations within the cell membrane of eukaryotic cells due to their role as component lipids of sphingomyelin. Thus, eventual changes in one of these components may affect or be an outcome of their disturbances. SM distribution on the outer surface of the cell membrane and on serum lipoprotein levels reflects the state of the cell membrane. Hori et al.^21^ found that differences in SM serum composition may have a significant physiological impact on cell fluidity due to lipid serum-cell membrane exhaustion. It was already suggested that a reduction in fluidity of cell membranes affects hemolysis and white blood cell migration due to decreased blood cell deformability^22^. Additionally, Hori et al.^21^ suggested that membrane fluidity is lower in patients with hematologic malignancies and that many SM species show concentration-dependent correlations with LDL in hematologic malignancies. This seems to be in accordance with our finding of decreased SMs in AML patients due to its increased uptake.

However, the most affected in AML patients compared to controls was sterol sulfate (ST 21:1;O2;S) by 259%. One of the roles of ST in cells is regulation of lipid metabolism, inflammatory responses and cell proliferation by correlation with cholesterol levels and depression of immune system responses by related mechanisms. Thus, the increased appearance in serum may indicate that tumor cells affect the regulation of lipid metabolism and the immune system to grow and survive, respectively. This seems plausible in accordance with Tatsuguchi et al.’s^22^ colon cancer research, which indicates that ST, a cholesterol sulfate, acts as an inhibitor of cell infiltration by effector T cells. There was also an increased presence of acylcarnitine (AC(8:0) by 87%) in serum. Simultaneously with STs, AC also plays a regulatory role in lipid metabolism, as it is broken down by plasma esterase present in blood to carnitine, which is used to transport FA into cells. Consequently, its elevated abundance seems to be correlated with tumor proliferation.

Additionally, we observed an abundance increase of over 100% of amino-hexadecanoic acid, which belongs to the lipoamino acid family. However, the main issue in the analysis of lipoamino acids is related to the low levels at which they occur naturally, and there is a concern that high results might be obtained because of the physiological effects of sampling methods^23^. Moreover, this is the first report on this topic in AML analysis. In eukaryotic cells, lipoamino acids are minor but ubiquitous components of cell membranes. Due to FA derivatives, their increased abundance may be correlated with tumor cell proliferation and increased production of their components, which eventually may not be anchored to the cell membrane for various reasons.

Interestingly, it was also noted that there was an increase in cortisol levels, a vital hormone originating from the human adrenal cortex. Sakhnevych et al*.*^24^ revealed that cortisol initiates LPHN1 expression at the transcriptional level, prompting translation within human AML cells (Fig. 4). Notably, cortisol lacks this effect in healthy leukocytes. This cascade results in galectin-9 secretion, safeguarding AML cells from immune response by NK cells or cytotoxic T cells. Thus, elevated levels of cortisol in AML patients indicate a potential for immune evasion.

In our study, we conducted an analysis of metabolomic changes within a post-treatment cohort, stratifying patients into responders and non-responders. Our primary objective was to uncover disparities in metabolomic profiles, potentially offering a novel means of assessing the efficacy of post-induction therapy. Notably, our investigation directed our attention towards alterations in glycerophospholipid metabolism, specifically focusing on SMs that displayed heightened abundance. This finding aligned with prior research^25,26^. Glycerophospholipid metabolism modifications have attracted significant attention due to their profound implications in the landscape of tumor progression and development. In the context of highly proliferating cancer cells, there is a demand for the de novo synthesis of fatty acids, a process vital for the continuous generation of glycerophospholipids essential for membrane synthesis. Glycerophospholipids hold a pivotal position in a range of cellular functions. These functions encompass not only the structural shaping of cell membranes but also their involvement in intricate signaling pathways^27^. Elevated serum glycerophospholipids could signal effective therapy-induced cancer cell elimination, causing the release of lipids from cell membranes.

We performed additional data analysis to research whether there is any outcome disparity between DAC versus DA regimens for patients with CR. However, we were unable to observe any statistically significant differences in the metabolome of patients with remission according to the different regimens. On the other hand, there is a single study showing the possible impact of cladribine on metabolomics changes, mostly in CLL patients (metabolic response patterns of nucleotides in B-cell chronic lymphocytic leukemia to cladribine, fludarabine and deoxycoformycin), which showed a possible determination of “*metabolic response patterns*'' of nucleotides in CLL cells treated with drugs that might distinguish patients with susceptible and refractory CLL prior to chemotherapy^28^.

A previously not researched topic, namely, the metabolomics differences in patients stratified based on the 2017 ELN risk classification, was investigated in the current study. The comparisons were performed before starting therapy; thus, the results represent the metabolome unaffected by medications. ELN classification stratifies patients into three risk categories (1—favorable, 2—intermediate, and 3—adverse) by refining the prognostic value of specific genetic mutations in comparison to the previous classification from 2010^29^. The first comparison (2 vs. 1) presented a slight change in PCs with a decreased abundance of up to 61%. However, the second comparison (3 vs. 1) represented a more vast library of disturbed metabolites compared to the highest and lowest risk stratifications. In addition to other glycerophospholipid metabolism compounds being mostly decreased, PE (16:0/20:5) was elevated by 360%, as well as in the first comparison where the same compound was increased by 226%. However, in the third comparison (3 vs. 2), PE (16:0/20:5) was not significant. This may indicate that PE (16:0/20:5) may be used as a discriminating biomarker between the first and other risk factor groups. PE (16:0/20:5) was not previously described as a biomarker in AML or in any other study. We were also able to observe an alpha-amino fatty acid of chain length C16, a 2-aminohexadecanoic acid, by analyzing the third comparison (3 vs. 2), which is the only significantly changed compound that may be discriminative between both higher-risk-stratified groups of patients. In conclusion, observations of these results present three discriminative compounds able to stratify patients based on EML risk classification just by serum metabolomics analysis.

Many genetic aberrations are intragenetic and can only be detected using molecular biology techniques. Internal duplication of the FLT3-ITD gene occurs in approximately 23% of patients with a normal karyotype. Thus, our aim was to research differences in the metabolomes of patients with or without FLT3 mutation, which in the future may contribute to the discovery of new biomarkers or therapeutic approaches. In both comparisons, FLT3-ITD/FLT3-WT and FLT3-ITD/Controls, the compounds were mostly SMs, PCs and LPCs involved in glycerophospholipid metabolism. Interestingly, in FLT3-ITD/FLT3-WT comparison, SM(d33:1) stood out the most (decreased by 80.3%) and appeared already in the overall discrimination between case and control, but not as much decreased (by 67%), indicating a slightly more discriminative manner in the presence or absence of mutation if disease is diagnosed. However, some studies on pediatric patients’ plasma and cell samples state that FLT3-ITD does not define the global leukemia metabolome but points to multiple metabolic feature disturbances, such as nucleosides, such as an increased abundance of guanine, hypoxanthine, inosine, adenosine, guanosine, and adenosine 5′-monophosphate and a decrease in tryptophan and 5-formyl-hydroxykynurenine^30^. This observation also corresponds to the findings of Lo Presti et al.^4^. In their study, they effectively differentiated between the FLT3-ITD and FLT3-WT subgroups. This distinction was attributed, among other factors, to a significant increase in choline levels among FLT3-ITD patients compared to those with FLT3-WT. The conversion of choline into PC contributes to the rise in PC levels, and this escalation is pivotal in facilitating cell transformation and promoting cancerous proliferation. Our results indicated increased levels of PC (40:5) by almost 51%. However, this result was not significant after *p*-value correction (Supplementary Table 7).

Additionally, we were not able to detect statistically significant ceramides in the serum metabolome. This result seems to be in accordance with the results of Dany et al.^31^, in which the presence of FLT3-ITD mutation suppresses ceramide generation and FLT3-ITD inhibition mediates ceramide-dependent mitophagy, leading to AML cell death. These findings combined may aid in composing and introducing new treatment approaches for AML patients with FLT3-ITD mutations, in which metabolomics has already been introduced in novel research on gilteritinib^32^, FLT3-Inhibitor AC220 (quizartinib) and the Complex I Inhibitor IACS-010759^33^ or analyzing resistance to sorafenib^34^.

Core binding factor acute myelogenous leukaemia (CBF-AML), comprising up to 12–15% of all AML cases^35^ characterised by the presence of either t(8;21) (q22;q22) or inv(16) (p13q22)/t(16;16), is considered good-risk AML in the context of cytarabine based intensive chemotherapy^36^. Compared with other cytogenetic AML groups, patients with CBF-AML are considered a favorable AML risk group based on their high remission rate and survival probabilities. However, approximately one-half of patients with CBF-AML are still not cured, there is a need for markers to identify patients unlikely to respond to current treatment and to develop novel therapeutic approaches based on a better understanding of the pathophysiology of the disease^35^. The general consensus is to reserve allogeneic Stem Cell Transplantation (SCT) for relapsed CBF AML. However, an argument can be made for SCT in the first remission for patients with suboptimal MRD clearance (minimal residual disease), where the expected risk of relapse might be high^37^. Our results indicate PC (34:5) and LPC (22:0), as the most discriminative factor in the case of the metabolomics approach, a glycophospholipids (Fig. 3B).

The MLL (mixed-lineage leukemia) gene, located on chromosome 11q23, is involved in chromosomal translocations in a subtype of acute leukemia, which represents approximately 10% of acute lymphoblastic leukemia and 2.8% of acute myeloid leukemia cases^38^. Despite modern chemotherapeutic interventions and the use of hematopoietic stem cell transplantations, infants, children, and adults with MLL-r leukemia generally have a poor prognoses and response to these treatments. Based on the frequency of patients who relapse, do not achieve complete remission, or have brief event-free survival, there is a clear clinical need for a new effective therapy^39^. Similarly to CBF, also glycerosphingolipids and sphinolipids seem to stand out the most in discriminative analysis (Fig. 3C).

The need for discriminative factors for CBF and new treatment targets for MLL could be explored through glycoproteomic research. Glycosylation, which starts in the endoplasmic reticulum (ER) and extends to the Golgi apparatus, is connected to protein glycosylation and lipid metabolism. Lipid synthesis and transport occur in the ER, with glycerophospholipids playing roles in cancer processes like growth, migration, and evading apoptosis, as well as in cell communication. Despite being distinct processes, glycosylation and lipid metabolism interact, with glycotransferases affecting function. Recent research links glycosylation and lipid metabolism to ER protein quality control, suggesting impacts on phospholipid levels and ER stress^40^. Sphingolipids, altered in our results, and glycoproteins are unrelated but might influence each other. Glycosidases, involved in glycoprotein breakdown, are linked to sphingolipid degradation. For instance, beta-galactosidase plays a role in sphingolipid and glycoprotein processing, possibly affecting glycosphingolipid-based signaling units. Enzymatic hydrolysis products might differ from the original functions^41^.

As stated above, metabolomics in AML screening has recently gained attention in research for new biomarkers or therapeutic targets. However, it is still poorly investigated, and in this work, we covered a new topic of unresearched differences in the metabolomes of AML patients. The differences may guide further research of new therapeutic approaches based on differences between patients stratified by risk ELM classification and in those with or without FLT3 mutation, CBF-AML, and MLL. Additionally, this study not only expands our knowledge of biology and metabolic pathways regarding metabolomics differences between patients with CR and NR but also guides further researchers in applying metabolomics as an aid in initial diagnosis or as a platform for identifying therapy targets.

### Supplementary Information

Supplementary Figure 1.

Supplementary Figure 2.

Supplementary Figure 3.

Supplementary Figure 4.

Supplementary Figure 5.

Supplementary Figure 6.

Supplementary Figure 7.

Supplementary Figure 8.

Supplementary Figure 9.

Supplementary Information.

Supplementary Tables.

## Data Availability

The datasets generated during and/or analyzed during the current study are not publicly available due to containing sensitive patient data but are available from the corresponding author on reasonable request.
