# Supplementary figures and images for: Metabolomic profile of acute myeloid leukaemia parallels of prognosis and response to therapy
Source: Sci Rep. 2023 Dec 9;13:21809. doi: 10.1038/s41598-023-48970-0 (PMC10710498; doi:10.1038/s41598-023-48970-0)

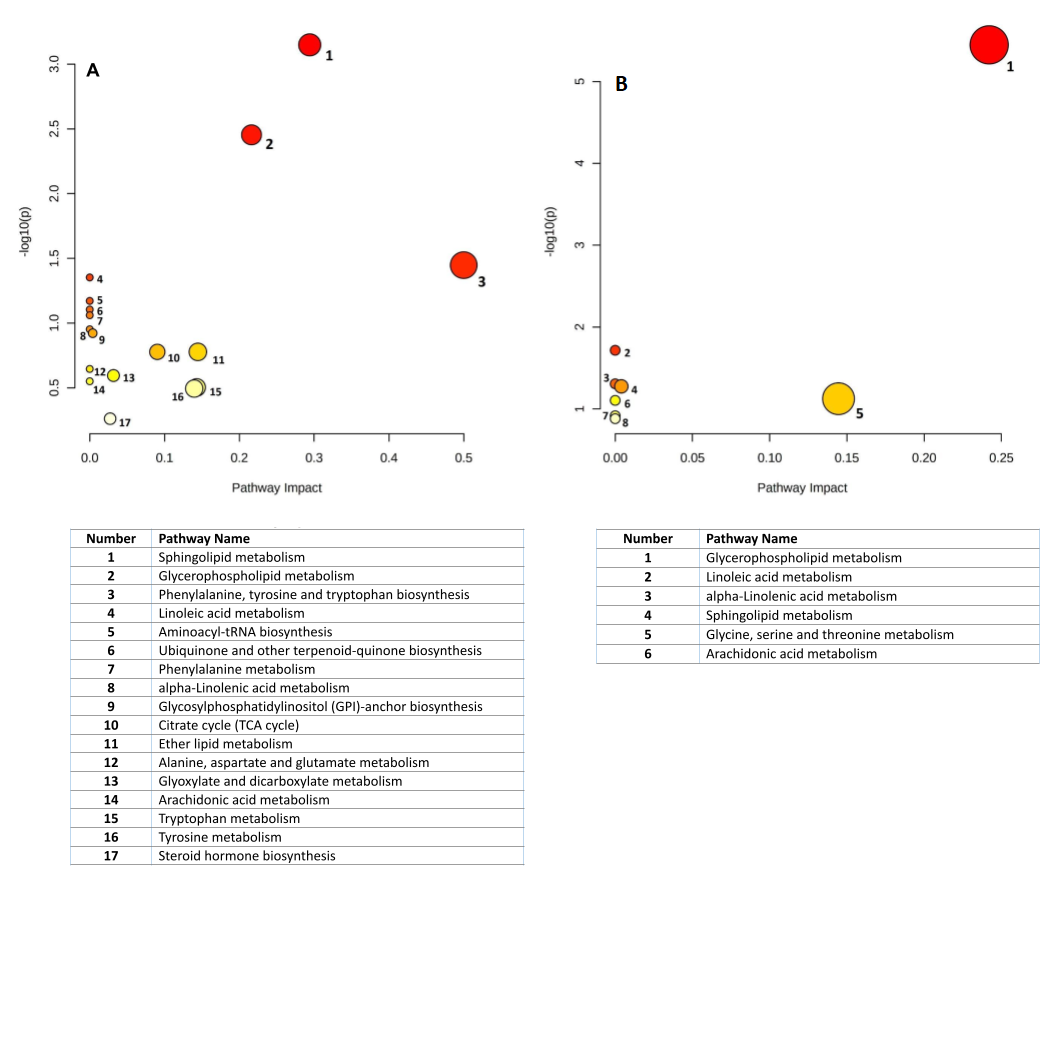

Supplement: Supplementary file 1 — Supplementary Figure 1. [file 41598_2023_48970_MOESM1_ESM.png]

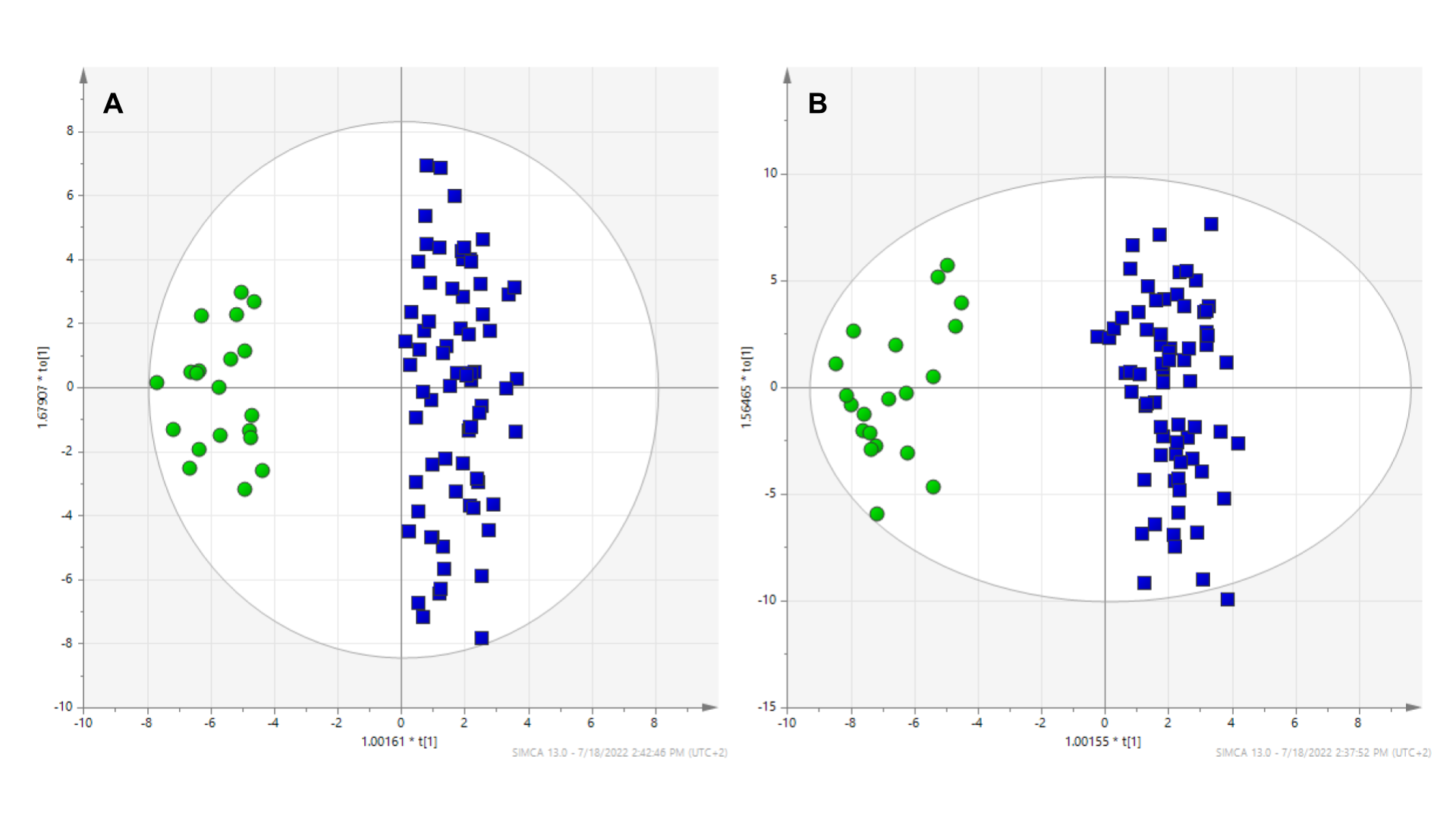

Supplement: Supplementary file 2 — Supplementary Figure 2. [file 41598_2023_48970_MOESM2_ESM.png]

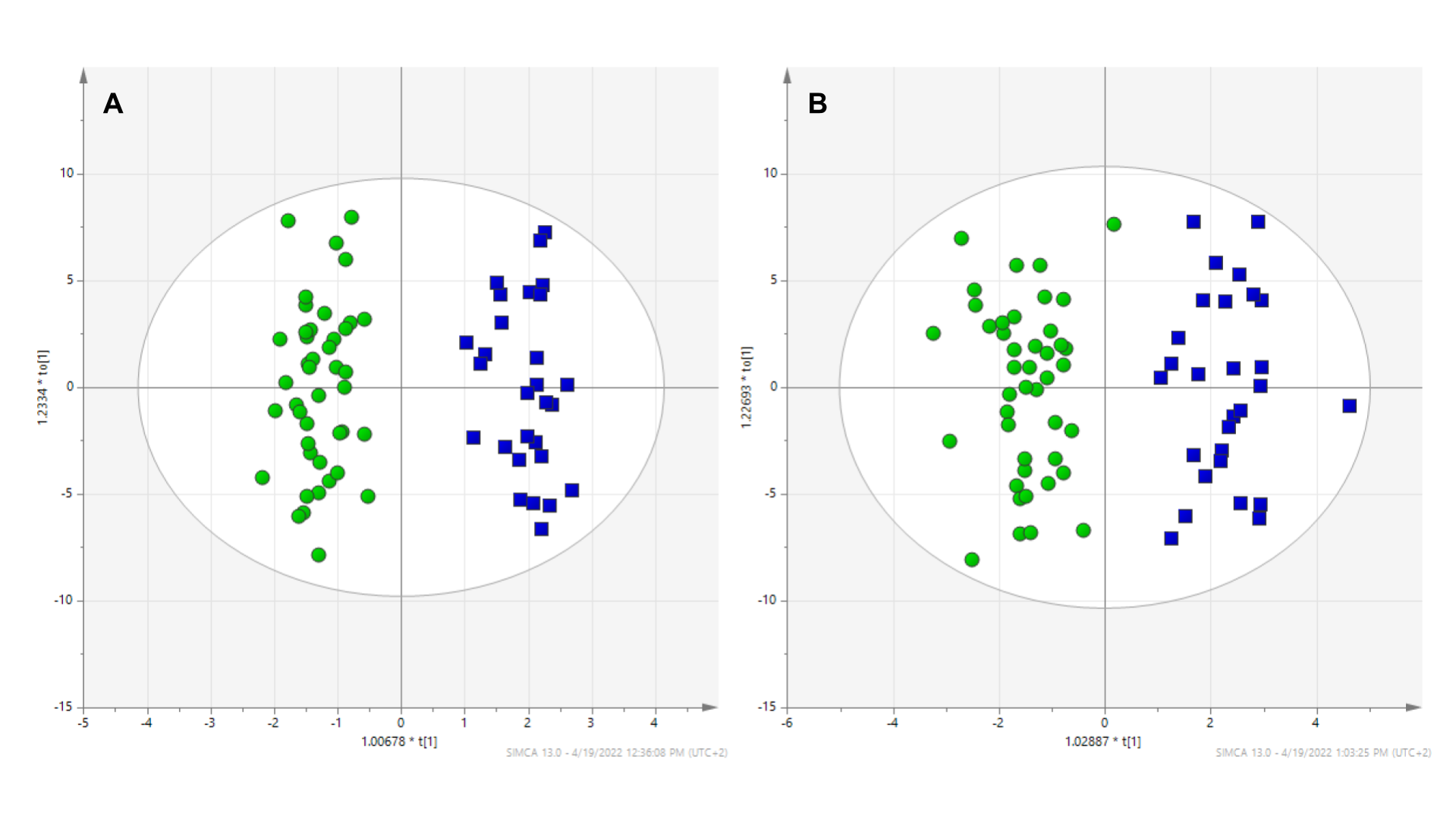

Supplement: Supplementary file 3 — Supplementary Figure 3. [file 41598_2023_48970_MOESM3_ESM.png]

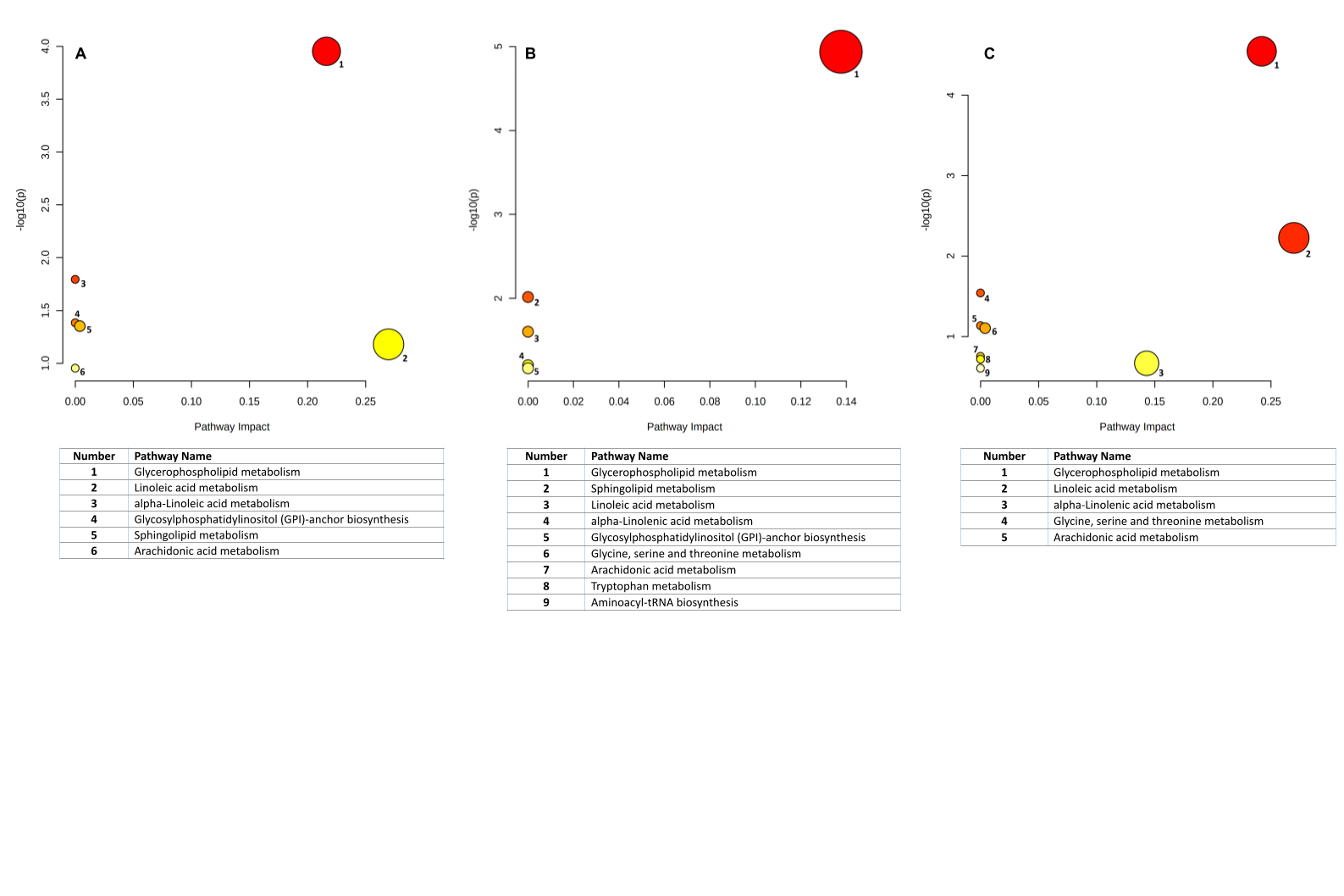

Supplement: Supplementary file 4 — Supplementary Figure 4. [file 41598_2023_48970_MOESM4_ESM.png]

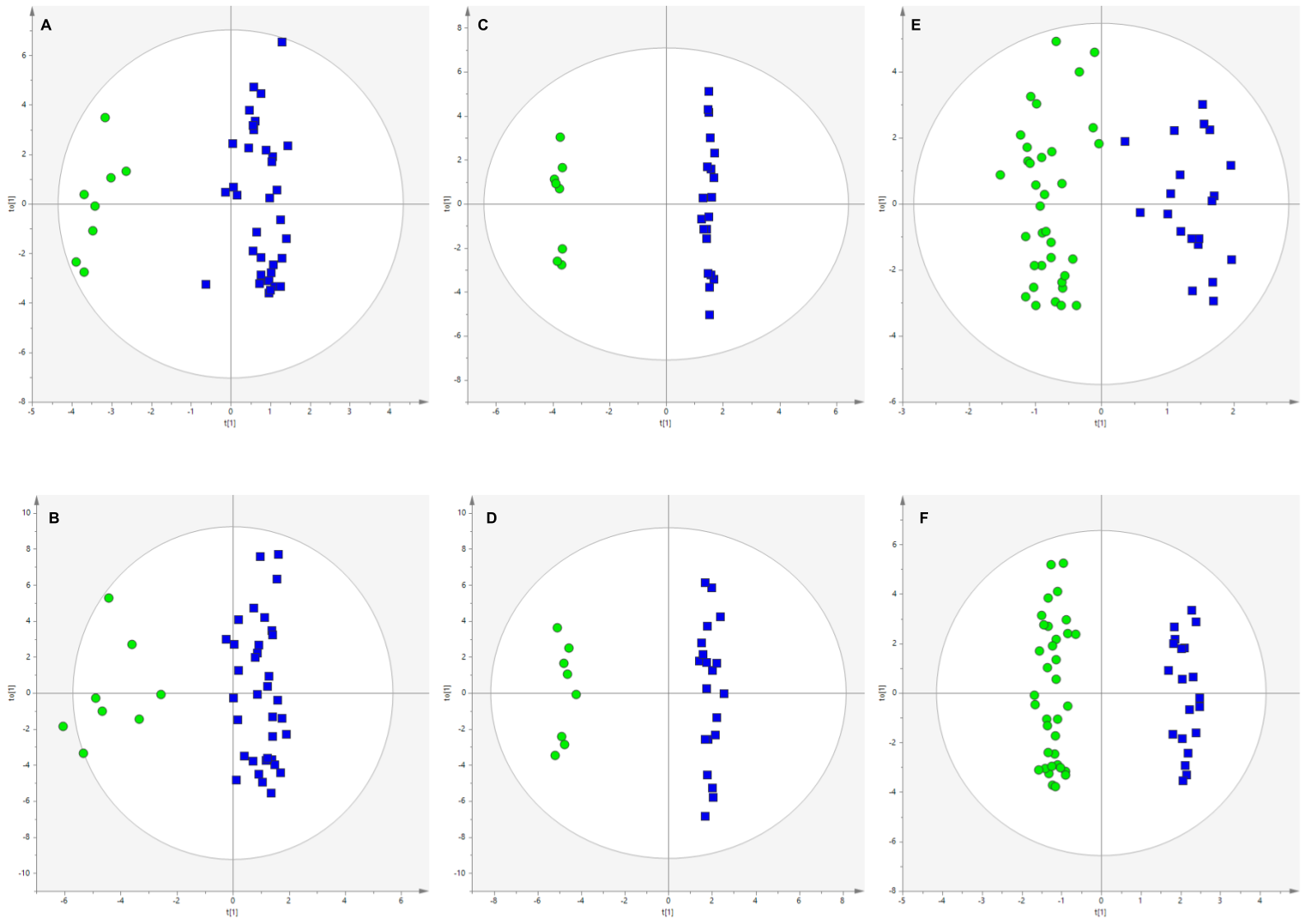

Supplement: Supplementary file 5 — Supplementary Figure 5. [file 41598_2023_48970_MOESM5_ESM.png]

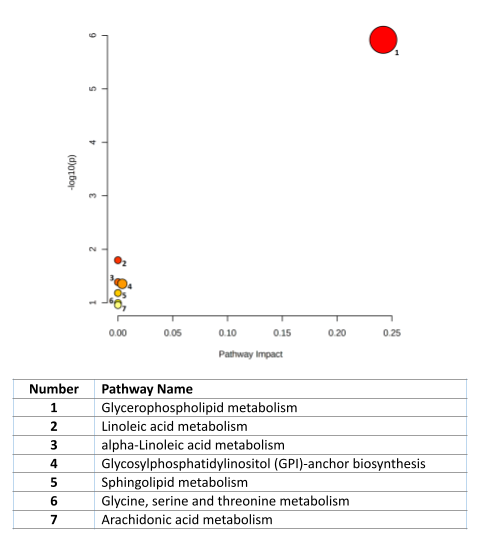

Supplement: Supplementary file 6 — Supplementary Figure 6. [file 41598_2023_48970_MOESM6_ESM.png]

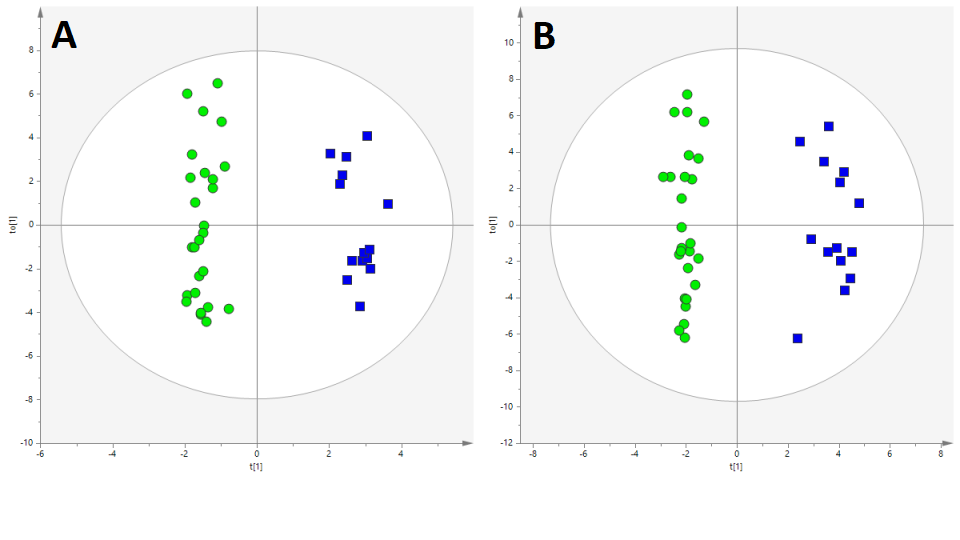

Supplement: Supplementary file 7 — Supplementary Figure 7. [file 41598_2023_48970_MOESM7_ESM.png]

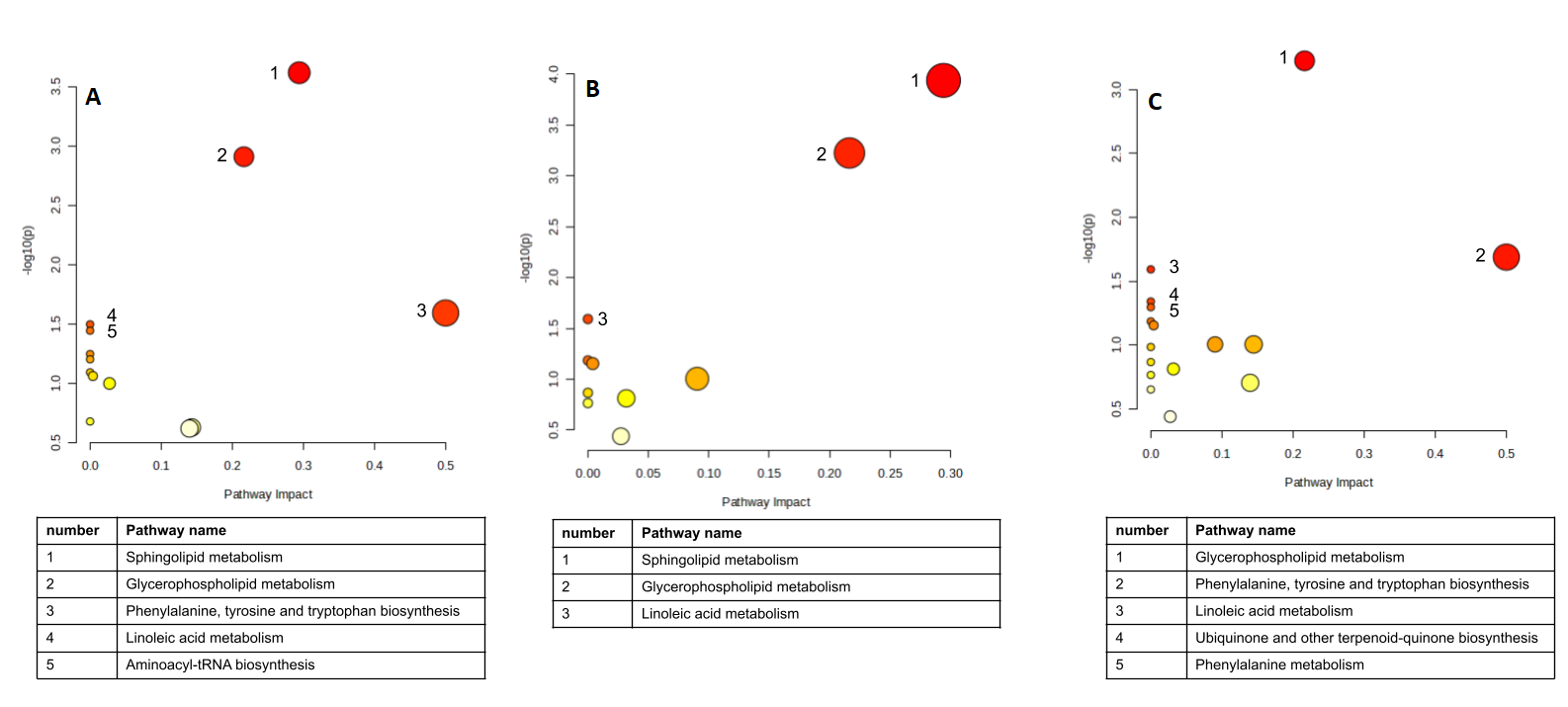

Supplement: Supplementary file 8 — Supplementary Figure 8. [file 41598_2023_48970_MOESM8_ESM.png]

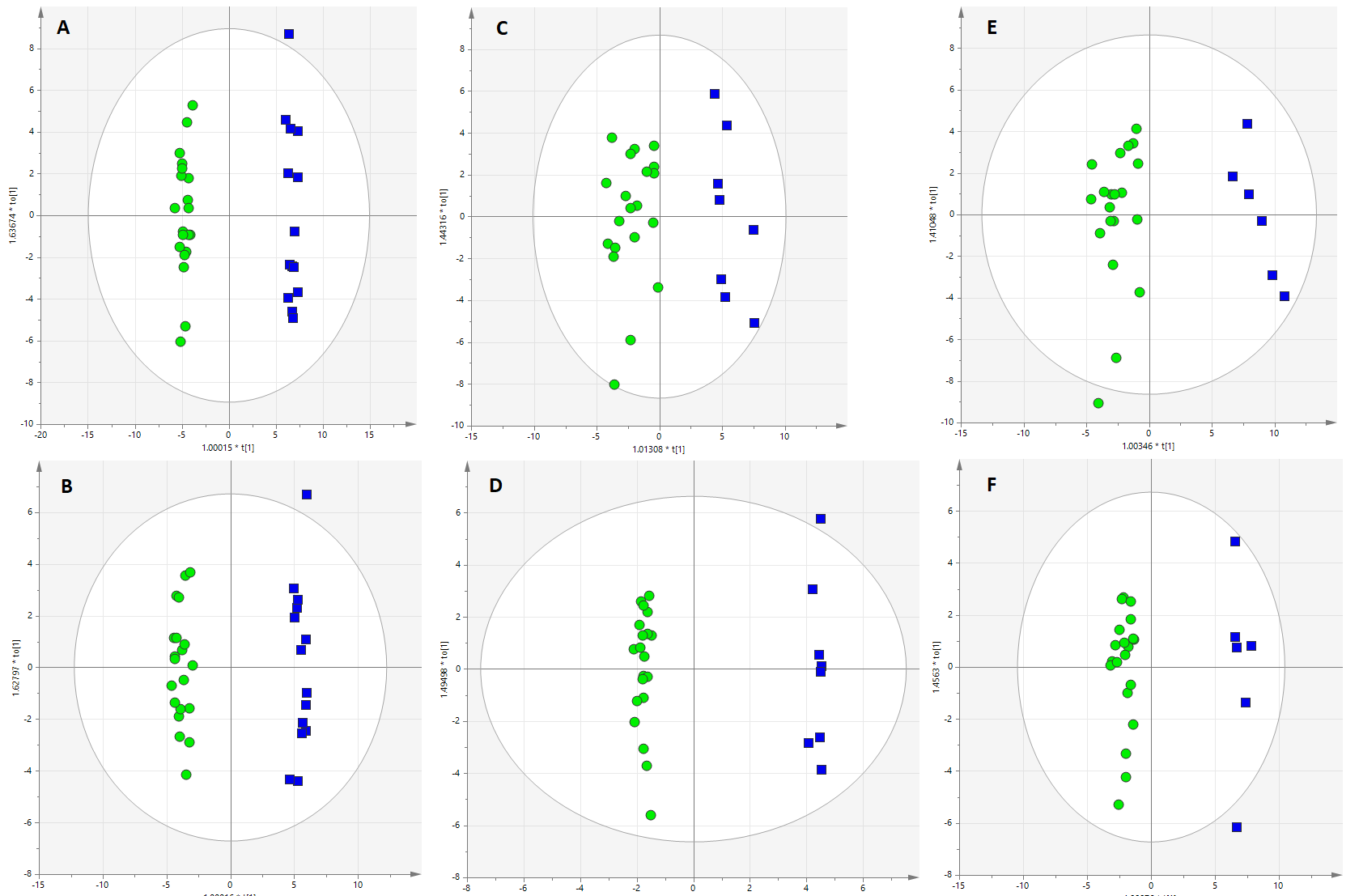

Supplement: Supplementary file 9 — Supplementary Figure 9. [file 41598_2023_48970_MOESM9_ESM.png]
