# Supplementary material for: Metabolomic profile of acute myeloid leukaemia parallels of prognosis and response to therapy
Source: Sci Rep. 2023 Dec 9;13:21809. doi: 10.1038/s41598-023-48970-0 (PMC10710498; doi:10.1038/s41598-023-48970-0)
Supplement: Supplementary file 10 — Supplementary Information. [file 41598_2023_48970_MOESM10_ESM.docx]

**Chemicals and reagents**

The Milli-Q Integral 3 system (Millipore SAS, Molsheim, France) was used to obtain purified water. Zomepirac sodium salt (used as the internal standard [IS]), LS-MS-grade acetonitrile, methanol, formic acid, and LC-grade ethanol were purchased from Sigma-Aldrich Chemie GmbH (Steinheim, Germany). The API-TOF reference mass solution kit (G1969-850001)
 and tuning solutions, ESI-L low-concentration tuning mix (G1969-85000), and ESI-TOF Biopolymer Analysis reference masses (G1969-850003) were purchased from Agilent Technologies (Santa Clara, CA, USA).

**Sample treatment and analysis**

Clotting Activator vacuum system tubes (2.4-mL) were used to collect whole blood which enabled us to obtain serum samples. In order to permit the formation of a clot within a 60 minutes period, the tubes were kept in the vertical position at room temperature. A centrifuge was used in a horizontal rotor (swing-out head) for a duration of 10 minutes at 1300 *g,* at room temperature, when the clotting had ended. Eppendorf tubes were used to collect serum fractions (0.5 mL each) and then stored at the temperature of –80°C until the day of analysis.

The thawing process was performed on ice on the day of the analysis. We performed vortex-mixing (for 1 minute) using 1 volume of the serum sample with 4 volumes of freezing cold (–20°C) methanol/ethanol (1:1) mixture containing 1 ppm zomepirac in order to extract metabolites and conduct protein precipitation. Subsequent to extracting the samples they were stored on ice for 10 minutes and consequently they were centrifuged at 21,000×*g* for 20 minutes at 4°C. 0.22-μm nylon filter was used to filter the supernatant into glass vials. In order to obtain quality control (QC) samples, an equal volume of all samples were mixed. The same procedure, which was applied for other samples, was used for the obtained mixture.

Liquid chromatography coupled with mass spectrometry (LC-MS) system consisting of 1290 Infinity I LC with a degasser, 2 binary pumps and a thermostated autosampler coupled to a 6545 Q-TOF-MS detector (both Agilent Technologies, Santa Clara, CA, USA) was used to randomly analyze samples. Positive (ESI+) and negative (ESI-) ion modes were used to perform analyzes, whereby 1 μL of the sample was injected into a thermostated (60°C) Zorbax Extend- C18 RRHT (2.1×50 mm, 1.8-μm particle size, Agilent Technologies) chromatographic column. The applied flow rate was 0.6 mL/min
 with solvent A (water with 0.1% formic acid) as well as solvent B (acetonitrile with 0.1% formic acid). The starting point of the chromatographic gradient was 5% of phase B for the first minute, consequently it was increased to 80% (from 1 to 7 minutes) and to 100% (from 7 to 11.5 minutes). When the gradient reached 100%, it returned to initial conditions (5% phase B) in 0.5 minutes, which was maintained from 12 to 15 minutes. Full scan mode from mass (m/z) 50–1000 was operated by the mass spectrometer. Applied capillary voltage for positive and negative ionization modes was set to 3 kV and 4 kV, respectively. The used nozzle voltage was at the level of 1000 V. For positive and negative ionization modes applied fragmentor voltage was 175 V and 200 V, respectively. We set the flow rate of drying gas at 12 L/min at 250°C and the gas nebulizer at 52 psig. Centroid mode at a scan rate of 1.5 scans per second was used to collect the data. Calibrant solution delivery along with a dual nebulizer ESI source enabled us to obtain accurate mass measurements. An isocratic pump (Agilent, Santa Clara, CA, USA) at a flow rate of 0.5 mL/min (1:100 split) facilitated to continuously introduce a calibrating solution containing reference masses at m/z 121.0509 (protonated purine) and m/z 922.0098 (protonated hexakis [1H,1H,3H-tetrafluoropropoxy] phosphazene or HP-921) in positive ion mode or m/z 119.0363 (proton abstracted purine) and m/z 966.0007 (formate adduct of HP-921) in negative ion mode.

**LC-MS data treatment**

MassHunter Qualitative Analysis Software (B.07.00, Agilent, Santa Clara, CA, USA) enabled us to clean the background noise and unrelated ions from the raw data gathered by the analytical instrumentation using the molecular feature extraction (MFE) tool. A list of all possible components described by mass, retention time (RT), and abundance was created by the MFE. For positive
 and negative ion mode the limit for the background noise for data extraction by MFE was set to 2000 and 1000 counts, respectively. We applied such adduct settings as +H, +Na, +K in positive ion mode
 and −H, +HCOO, +Cl for negative ion mode in order to identify co-eluting adducts of the same feature. Both ionization modes could be used to observe neutral dehydration losses. Mass Profiler Professional 15.1 (Agilent, Santa Clara, CA, USA) was used to perform sample alignment and data filtering.
 For the alignment, we used such parameters as 1% for RT and 15 ppm for the mass variation. For further data treatment, we decided to keep only those metabolic features that were detected in >50% in QC samples with the coefficient of variation (CV) <20%. Additionally, the features were filtered to keep only those present in at least 80% of the samples in at least one of the studied groups in each comparison independently. Missing values were replaced as described by Armitage et al. (2015).

**Metabolite identification**

Tandem mass spectroscopy (MS/MS) fragmentation was the basis of identification of metabolites. The METLIN, KEGG, LIPIDMAPS, and HMDB databases, which were simultaneously accessed by CEU Mass Mediator (http://ceumass.eps.uspceu.es/mediator) were used to search for accurate masses of features. We confirmed the identity of metabolites by matching the experimental MS/MS spectra to MS/MS spectra from databases of fragmentation spectra and retention time obtained for the metabolite’s standard. Identical chromatographic conditions for the primary analysis were used repeatedly to perform the experiments. Ions were targeted for collision-induced dissociation (CID) fragmentation on the fly based on the previously determined accurate mass and retention time. Previously described characteristics of the fragmentation pattern were used to identify phospholipids. Supplementary Table 1 presents characteristic fragments of identified metabolites [S.Tab.1].

**Statistical analysis**

In order to evaluate data quality, multivariate statistics were used. It was achieved by checking the location of the QC samples on principal component analysis (PCA) plots. It also helped to observe sample discrimination on orthogonal partial least squares discriminant analysis (OPLS-DA) plots. SIMCA−P+ 13.0.3.0 (Umetrics, Umea, Sweden) was used to perform multivariate calculations and plots. The Mann-Whitney nonparametric U-test with the Benjamini-Hochberg false discovery rate (FDR) correction was used for univariate statistics. The level of statistical significance was at the level of 95% (P < 0.05). Univariate statistics was performed in Mass Profiler Professional 15.1 (Agilent, Santa Clara, CA, USA). The t-test, Metabolic pathway analysis as well as Biochemical Importance Plots were performed in MetaboAnalyst 5.0.

**Supplementary information**

**Supplementary Fig. 1. Metabolomic Pathway Analysis** of metabolites contributing to group separation between: **A.** AML patients and controls. Metabolic pathways differentiating AML patients from healthy control showed the greatest variety in sphingolipid, glycerophospholipid and amino acid metabolism. **B.** CR and NR. Metabolic pathways differentiating CR patients from NR patients showed the greatest variety in glycerophospholipid and linoleic metabolism. The identified pathways are visualized as circular shapes, with their colors determined by p-values (with darker shades indicating more pronounced alterations in associated metabolites). Additionally, the size of each circle represents the pathway's impact score. The pathways with the greatest impact, supported by substantial statistical significance scores, are highlighted with annotations.

**Supplementary Fig. 2. Multivariate analysis of AML serum LC‒MS metabolomics data.** An orthogonal partial least squares discriminant analysis (OPLS-DA) plot showed clear separation between AML patients and controls: **A.** in negative ionization mode; R^2^ = 0.920, Q^2^ = 0.764. **B.** in positive ionization mode; R^2^ = 0.926, Q^2^ = 0.764.

**Supplementary Fig. 3. Multivariate analysis of AML serum LC‒MS metabolomics data.** An orthogonal partial least squares discriminant analysis (OPLS-DA) plot showed clear separation between AML patients with CR and NR. **A.** in negative ionization mode; R^2^ = 0.940, Q^2^ = 0.511. **B.** in positive ionization mode; R^2^ = 0.875, Q^2^ = 0.550.

**Supplementary Fig. 4. Metabolomic Pathway Analysis** of metabolites contributing to group separation between 2017 ELM risk-stratified patients. Pathway analysis of metabolites significantly associated with differences in serum between: **A.** 2 to 1. Metabolic pathways differentiating intermediate risk category from favourable risk category showed the greatest variety in glycerophospholipid, and linoleic acid metabolism. **B.** 3 to 1. Metabolic pathways differentiating adverse risk category from favourable risk category showed the greatest variety in glycerophospholipid metabolism. **C.** 3 to 2. Metabolic pathways differentiating the adverse risk category from the intermediate risk category showed the greatest variety in glycerophospholipid, linoleic acid, and alpha-linoleic acid metabolism. The identified pathways are visualized as circular shapes, with their colors determined by p-values (with darker shades indicating more pronounced alterations in associated metabolites). Additionally, the size of each circle represents the pathway's impact score. The pathways with the greatest impact, supported by substantial statistical significance scores, are highlighted with annotations.

**Supplementary Fig. 5. Multivariate analysis of AML serum LC‒MS metabolomics data of risk-estimated patients.** An orthogonal partial least squares discriminant analysis (OPLS-DA) plot showed clear separation between **A.** 2 and 1 risk-estimated patients in negative ionization mode; R^2^ = 0.931, Q^2^ = 0.640. **B.** 2 and 1 risk-estimated patients in positive ionization mode; R^2^ = 0.903, Q^2^ = 0.593. **C.** 3 and 1 risk-estimated patients in negative ionization mode; R^2^ = 0.998, Q^2^ = 0.792. **D.** 3 and 1 risk-estimated patients in positive ionization mode; R^2^ = 0.991, Q^2^ = 0.766. **E.** 3 and 2 risk-estimated patients in negative ionization mode; R^2^ = 0.894, Q^2^ = 0.723. **F.** 3 and 2 risk-estimated patients in positive ionization mode; R^2^ = 0.979, Q^2^ = 0.820.

**Supplementary Fig. 6. Metabolomic Pathway Analysis** of metabolites contributing to group separation between patients with FLT3-ITD. Pathway analysis of metabolites significantly associated with differences between FLT3-ITD and FLT3-WT AML patients serum. Metabolic pathways associated with FLT3-ITD showed the greatest variety in glycerophospholipid metabolism. The identified pathways are visualized as circular shapes, with their colors determined by p-values (with darker shades indicating more pronounced alterations in associated metabolites). Additionally, the size of each circle represents the pathway's impact score. The pathways with the greatest impact, supported by substantial statistical significance scores, are highlighted with annotations.

**Supplementary Fig. 7. Multivariate analysis of serum LC‒MS metabolomics data of patients with FLT3-ITD mutation and FLT3-WT.** An orthogonal partial least squares discriminant analysis (OPLS-DA) plot showed clear separation between AML patients with FLT3-ITD mutation and FLT3-WT: **A.** in negative ionization mode; R^2^ = 0.971, Q^2^ = 0.786. **B.** in positive ionization mode; R^2^ = 0.968, Q^2^ = 0.801.

**Supplementary Fig. 8. Metabolomic Pathway Analysis** of metabolites contributing to group separation between patients with distinct genetic subgroups from control group. **A.** FLT3-ITD to control group; **B.** CBF-AML to control group; **C.** MLL to control group. The identified pathways are visualized as circular shapes, with their colors determined by p-values (with darker shades indicating more pronounced alterations in associated metabolites). Additionally, the size of each circle represents the pathway's impact score. The pathways with the greatest impact, supported by substantial statistical significance scores, are highlighted with annotations.

**Supplementary Fig. 9. Multivariate analysis of AML serum LC‒MS metabolomics data.** An orthogonal partial least squares discriminant analysis (OPLS-DA) plot showed clear separation between AML patients between: **FLT3-ITD mutation and control group - A.** in positive ionization mode; R^2^ = 0.932, Q^2^ = 0.810. **B.** in negative ionization mode; R^2^ = 0.945, Q^2^ = 0.862; **CBF-AML and control group - C.** in positive ionization mode; R^2^ = 0.881, Q^2^ = 0.680. **D.** in negative ionization mode; R^2^ = 0.875, Q^2^ = 0.682; **MLL and control group - E.** in positive ionization mode; R^2^ = 0.937, Q^2^ = 0.795. **F.** in negative ionization mode; R^2^ = 0.955, Q^2^ = 0.795;
